# Our New York Letter

**Published:** 1891-04

**Authors:** 


### Correspondence.

### OUR NEW YORK LETTER.

NEW YORK, March 15, 1891.

The enthusiasm that had been evoked on this side of the water when the first announcement of the Koch cure for tuberculosis was made public, has completely petered out in this city and has left in its train nothing but blighted hopes, and more or less bankrupt fortunes. A number of physicians, carried away by the enthusiasm of the hour, started large and expensve sanitariums for the cure of tuberculosis after the most approved Koch fashions, and these sanitariums still exist as monuments to their mistaken zeal and want of better judgment.

But though the Koch remedy has proved utterly abortive in all forms of pulmonary tuberculosis, in lupus, however, signal success has been achieved, as has been prophetically stated to you in a previous letter. Dr. H. Holbrook Curtis, of this city, recently read a very interesting paper at the New York Academy of Medicine, entitled: "A Case of External Lupus and its Reactions, under Koch's Lymph, with Apparent Cure." The patient was a boy sixteen years of age who had a lupus for six years on his neck. He was treated at St. John's Riverside Hospital, Yonkers, N. Y., receiving the first inoculation of the lymph January 1st, 1891. The patient received in all, seven injections during a period of twenty days, with the characteristic reactions each time manifesting themselves. At the first injection, one milligramme of the fluid was given, and this was gradually increased to three, which was the largest amount that was injected at any one time. The seventh injection was given at 5 p. m., on the evening of the 23d of January, with no marked reaction in any way differing from the foregoing. Ten hours after the inoculation the portion of the scab that remained began to change in appearance, and on the morning of the 24th, the diseased tissue looked as though plaster of paris had been dusted over it. After each of the reactions, those portions of the scab that had turned white dropped off, leaving behind a healing surface, an apparent cure having been effected.

Dr. T. Gaillard Thomas has been again induced to return for a brief period to the scene of his former labors and achievements. He has just closed a series of four lectures on the pathology and treatment of certain forms of uterine disease, and treated the subject in his usually lucid and interesting manner. He related a case of rare occurrence in gynecological practice, which may prove of some interest to your readers. Speaking of certain diseases peculiar to the menopause, he said that he had been recently called to see a lady sixty years of age for whom a convenient diagnosis of cancer had been made by three physicians of this city, based upon the following symptoms. She had stopped menstruating ten years previously, and as she was one day walking about the floor of her parlor suddenly a gush of fluid of a pinkish, watery character and disagreeable odor came from the vagina. The patient felt entirely relieved as a result of this explosion. Since that time, at regular intervals of two months, she has had a similar discharge.

Dr. Thomas was sent for by the husband and daughter of the old lady, to make an examination of the case. On examination of the abdomen he found by palpation a globular mass as large as a child's head. He put the patient under an anaesthetic, forced a uterine sound through the cervical canal; after first snipping the external os with scissors, he then passed a dilator through the canal. As he did this, about ten ounces of a dirty, pinkish fluid gushed out. He next took a curette and passed it over the entire lining membrane of the cavity of the uterus, scraping it with moderate force, thinking she had hydatids of that organ. It was found, however, entirely free from any such trouble. An aluminum stem was next passed through the canal and permitted to remain in situ.

The patient was entirely cured, as a result of these different manoeuvres. The diagnosis in this case was that of a uterine cavity distended by gas, a condition due alone to the change of life in women. This is a rare affection, but does, however, occasionally occur. It was explained on these grounds. This old lady had, at fifty years of age, a uterine catarrh invading the Fallopian tubes. The fluid secreted by the diseased mucous membrane of the uterus, had been pouring through the cervical canal. As the cervix closed on the fluid the air that had been contained in the cavity of the uterus set up a fermentative action, and as a result, there was a distention of the uterus by gas.

Dr. Ralph Walde, of the New York Post-Graduate School, said, recently, to the writer: "It is a well-known fact that experienced gynecologists have passed a sound into a gravid uterus, advised a pregnant woman to have a serious surgical operation performed upon her or pronounced a non-pregnant woman with child. These facts have led me to make a study of the early symptoms of pregnancy, and I am now convinced that in a large majority of cases, it is possible to make a diagnosis of pregnancy during the first six or eight weeks with as much certainty as one can make a diagnosis of pneumonia, when you hear the crepitant rale, or valvular disease of the heart when a mitral murmur is present. All the well known symptoms should be taken into account when you are able to elicit them from the patient, but many women have very few, if any, rational symptoms, during the early stage of pregnancy. For the past two or three years I have principally depended upon Hegar's sign, as described by Dr. E. H. Grandin. This sign has never been found where pregnancy did not exist, nor have I ever failed to find it where the body of the uterus could be examined by bi-manual palpation. In the early stage of pregnancy a condition of active hyperemia exists in the uterus, and with the exception of a very slight softening at the external, as these changes are principally confined to the body of the organ, causing an enlargement in its transverse diameter, especially the antero-posterior, which is made to project over the cervix. The whole organ under these circumstances very much resembles a fat-bellied jug. As the enlargement in the corpus is due to an increase in the amount of arterial blood flowing through it, it is consequently resilient and this resiliency is my main guide in diagnosis. The examination is made in this manner: Place your left hand on the abdomen of the woman and your right index finger in the vagina, and as pressure is made against the corpus with the index finger it is bound to yield. As soon, however, as this pressure is removed the body again springs back to its former shape. In other words, the corpus is resilient.

A man aged 35, a painter by occupation, was operated upon at the New York Polyclinic, by Prof. J. A. Wyeth, by a novel procedure. He had an aneurism of the ascending portion of the aorta, which was as large as the head of a baby, and the result of a syphilis he had acquired some years previously. He was suffering at this time from a marked syphilitic dyscrasia and was put upon iodide of potassium, taking as large a dose as 350 grains of the drug a day. He improved somewhat under treatment, but the aneurism remained as large as before. Dr. Wyeth adopted the method recommended by Macewen, of Glasgow, and inserted four silver needles, sterilized by being boiled for from ten to fifteen minutes in water, into the cavity of the aneurism at different points. These were permitted to remain in situ for a couple of days, when they will be withdrawn and again iuserted at different point. This is done with the hope of inducing coagulation and formation of a white thrombus in the sac. The patient was seen yesterday and was making very favorable progress toward recovery. P. J. R.

---

CHILDREN THREATENED WITH CROUP.—

℞ Vini ipecac, ʒ iij.

Syr. tolu, ʒ v.

Mucil. Acaciae, ℥ j.

M. ft. mist. Sig. A small teaspoonful every hour or two.

*—Dr. Cheyne.*

---

A STIMULATING EXPECTORANT.—

℞ Ammon. carbonat., gr. v.
Tr. nuc. vomicae, mx.
Tr. scillae, ʒ ss.
Inf. serpentariae, ℥ j.

M. Sig. Three times a day.

*—Fothergill.*

---

SUB-ACUTE PLEURISY.—

℞ Pulv. Digital.,
Pulv. sallæ Mer,
Hydrarg chlor. mit., aa gr. x.

M. Et ft. pil. x et sig. One pill thrice daily.

*—Dr. Alonzo Clark.*

